# A Room-Temperature CNT/Fe_3_O_4_ Based Passive Wireless Gas Sensor

**DOI:** 10.3390/s18103542

**Published:** 2018-10-19

**Authors:** Tao Guo, Tianhao Zhou, Qiulin Tan, Qianqian Guo, Fengxiang Lu, Jijun Xiong

**Affiliations:** 1 Key Laboratory of Instrumentation Science & Dynamic Measurement, Ministry of Education, North University of China, Tai Yuan 030051, China; guotao6@nuc.edu.cn (T.G.); 18734580862@163.com (T.Z.); qqguo0214@163.com (Q.G.); peaceliveluna@163.com (F.L.); xiongjijun@nuc.edu.cn (J.X.); 2 Science and Technology on Electronic Test and Measurement Laboratory, North University of China, Taiyuan 030051, China

**Keywords:** wireless passive, carbon nanotube/Fe_3_O_4_ thin film, gas sensor, NH_3_

## Abstract

A carbon nanotube/Fe_3_O_4_ thin film-based wireless passive gas sensor with better performance is proposed. The sensitive test mechanism of LC (Inductance and capacitance resonant) wireless sensors is analyzed and the reason for choosing Fe_3_O_4_ as a gas sensing material is explained. The design and fabrication process of the sensor and the testing method are introduced. Experimental results reveal that the proposed carbon nanotube (CNT)/Fe_3_O_4_ based sensor performs well on sensing ammonia (NH_3_) at room temperature. The sensor exhibits not only an excellent response, good selectivity, and fast response and recovery times at room temperature, but is also characterized by good repeatability and low cost. The results for the wireless gas sensor’s performance for different NH_3_ gas concentrations are presented. The developed device is promising for the establishment of wireless gas sensors in harsh environments.

## 1. Introduction

As a highly toxic compound, ammonia (NH_3_) plays a vital role in all forms of life, and it is generally an outcome of natural processes in humans, animals, and plants. Ammonia is also the second most widely used chemical in the world [1,2]. Natural ammonia is present in the atmosphere at low ppb (1–5 ppb) levels. For humans, high concentrations of NH_3_ (ca. >300 ppm) may be damaging to the skin, eyes, and respiratory tract. In addition, NH_3_ is flammable at concentrations up to ca. 15–28% by volume in air [3]. Moreover, NH_3_ is widely used in various applications, including refrigeration, refining, manufacturing, cleaning, and nitrogenous fertilizers [4,5]. In order to protect workers’ health, the Occupational Exposure Limit (OEL) is set at 25 ppm of ammonia for an 8 h exposure and 35 ppm for a short-term exposure over 15 min [6]. In mines, the concentration of NH_3_ should not be higher than 0.004% (40 ppm), and the wired sensor will generate heat and may even produce electrospark, which is very dangerous in the mine. A wireless passive sensor can avoid the security threat caused by sensor heating and has no energy consumption. It also plays an important role when detecting gas in confined spaces [7]. A wireless sensor is usually installed easily and has a long service life. Therefore, it is necessary to design a wireless gas sensor that can measure NH_3_. In practical applications, there are many occasions where wireless gas sensors are needed, and the environment is usually harsh [8,9]. Ceramics have good mechanical properties, stable physical and chemical properties and do not easily be worn and corroded. Ceramics are suitable for various environments and can be good substrate materials. 

A great deal of research on NH_3_ sensors has been performed, considering inorganic, inorganic oxide/dioxide, and conducting polymers [10,11,12,13], which may be expensive, require a high temperature for operation, and are usually characterized by high power consumption and low sensitivity. Conventional materials such as metal oxides (SnO_2_, TiO_2_, MoO_3_, V_2_O_5_, and In_2_O_3_) require high operating temperatures, typically in the 200–500 °C range, which leads to significant power consumption [14,15,16]. As one of the most important metal oxides, Fe_3_O_4_ has a cubic inverse spinel structure and is one of the basic magnetic materials. However, like most metal oxide semiconductors, Fe_3_O_4_ has poor conductivity, which limits its ability to detect gases at room temperature [17]. Carbon nanotubes have good conductivity at room temperature, and the introduction of carbon nanotubes can improve the gas sensing properties of metal oxide semiconductors [18,19]. As a famous carbon-based material, carbon nanotubes (CNTs) are long, thin cylinders of carbon (typical diameter ranging from 1 nm to 100 nm) that are unique due to their size, shape, and physical properties. With the development of nanophase materials, CNTs have been recognized as one of the most promising and cost effective. CNTs are widely used in different gas sensors, owing to their excellent electronic and thermal properties, high surface-to-volume ratio, remarkable mechanical stiffness and excellent conductivity. CNTs are ideal for gas sensing, owing to their high surface absorption and significant conductivity change at room temperature [20,21,22,23], and are also used to detect NH_3_ [24,25,26]. To the best of our knowledge, there are few reports on gas sensors based on Fe_3_O_4_/CNT nanomaterials working at room temperature. Therefore, we tried to combine CNTs with Fe_3_O_4_ to develop a room temperature NH_3_ sensor. The proposed sensor demonstrates better performance than sensors based on pure CNTs or Fe_3_O_4_ films.

## 2. Sensor Testing Principle

### 2.1. Wireless Coupling Principle

**Figure 1:**
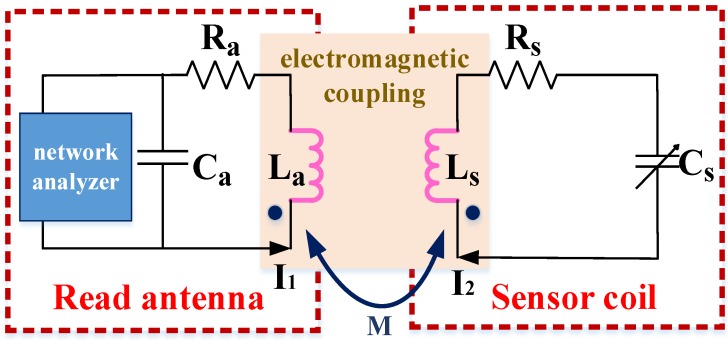
Sensor coupling model.

In 1999, English and Allen proposed a movable micromechanical ceramic wireless passive pressure sensor [27,28,29]. The wireless sensor designed in this paper is based on the principle of LC (Inductance and capacitance) resonance. Its electrical structure is a planar spiral inductor. Compared with the traditional LC resonant sensor composed of capacitance and inductance, the capacitance of the proposed sensor is the parasitic capacitance of the planar spiral inductor, and it has a simpler structure. Figure 1 shows the equivalent circuit model of the proposed sensor.

Figure 1 shows the electromagnetic coupling principle. A planar square spiral inductor ($L_{s}$) was printed on a ceramic substrate. Fe_3_O_4_/CNT thin films were coated on the spiral inductor for NH_3_ sensing. NH_3_ absorption by this sensor results in the charge transfer of the film and a change in the resistance of the LC sensor. At the same time, the self-resonant frequency ($f_{0}$) also changes, and $f_{0}$ can be obtained from Equation (1) [30]:

(1) $$\begin{array}{c} f_{0}=1.3996\times 10^{5}\frac{1}{P^{1.25}}-2.6489\ln P+1.9026\rho^{2}\\ +10^{-2}\left(5.7674S-4.512W\right)+25.062 \end{array}$$

In the above equation, P is the total length of the coil, S is the coil spacing, W is the coil width, and ρ is the fill factor that is given by Equation (2) [30]:

(2) $$\rho =\frac{D_{\mathrm{out}}-D_{\mathrm{in}}}{D_{\mathrm{out}}+D_{\mathrm{in}}}$$

In the above, $D_{\mathrm{out}}$ is the outer diameter of the coil, and $D_{\mathrm{in}}$ is the inner diameter.

The inductance of the inductor $L_{s}$ can be expressed using Equation (3) [30]:

(3) $$\begin{array}{c} L_{s}=10^{-3}\times N\times P(-3.0816-0.89529\ln\rho +6.7569\sqrt{\rho }\\ -4.7864\rho +1.8472\rho^{2}-00.53704\rho^{3}) \end{array}$$

In the above equation, N is the number of spiral inductor coils.

The parasitic capacitance of the sensor $C_{s}$ can be obtained from Equation (4) [30]:

(4) $$f_{0}=\frac{1}{2\pi \sqrt{L_{s}C_{s}}}$$

### 2.2. Gas Sensing Mechanism

**Figure 2:**
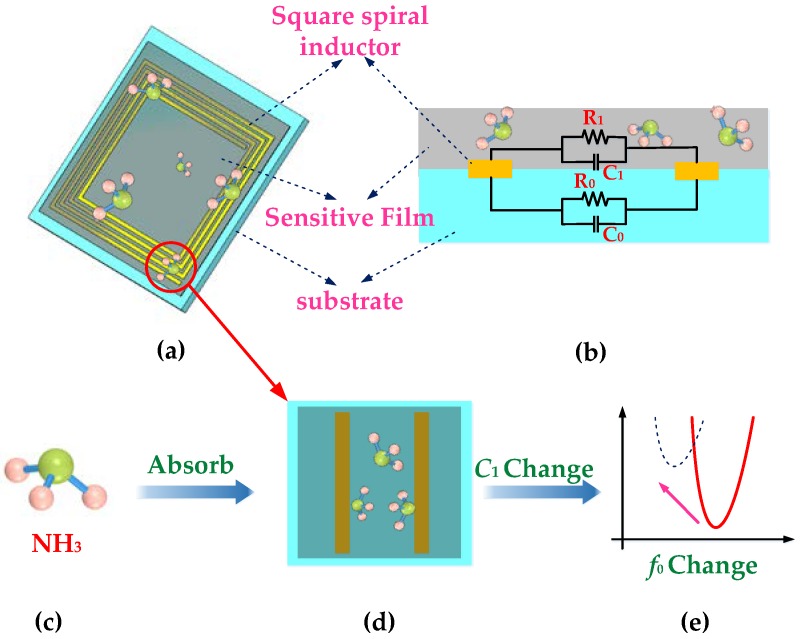
(**a**) Sensor model. (**b**) Sensor circuit model. (**c**) Ammonia molecule model. (**d**) Diagram of adsorbed gas molecules. (**e**) Trend of resonant frequency.

The gas molecules are usually adsorbed by the nanomaterials coated on the surface of the gas sensor [31]. In this process, the migration of electrons leads to the conductance change of the sensitive element, which reflects the corresponding change in the gas concentration [32]. In this paper, the change in NH_3_ concentration is mainly reflected in the variation of S_11_ parameters and resonance frequency. Apart from the change of the impedance of the sensor, external factors such as coupling distance also have a great impact on S_11_ parameters. In order to ensure the accuracy of the experiment, the resonant frequency, which is less affected by external factors, is selected to reflect the change of NH_3_ concentration [33]. By recording the resonant frequency, a change in the external gas concentration can be obtained. The mechanism of gas sensing is schematically shown in Figure 2. 

**Figure 3:**
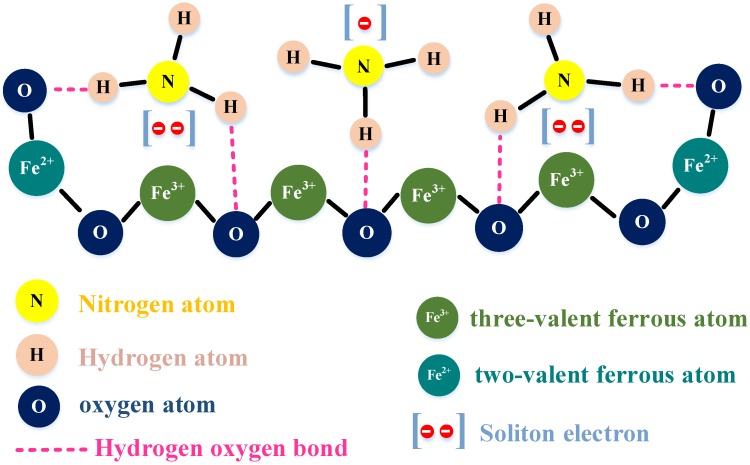
Model of the intermolecular binding force.

In the present design, ammonia was detected using a Fe_3_O_4_/CNT-based sensitive material. As a common nanomaterial, Fe_3_O_4_ is often used as a sensitive material for gas sensors, and the response of Fe_3_O_4_/CNTs obtained by doping is more intense due to the increase of electrical conductivity [17]. In this paper, the use of Fe_3_O_4_ was mainly based on its magnetic catalysis and chemical bonding. Fe_3_O_4_ consists of a trivalent iron atom and two-valent ferrous atoms. When the surface of the sensitive membrane is exposed to ammonia, the ammonia molecules can be adsorbed on the surface owing to the activity of Fe_3_O_4_, the N atoms in the ammonia gas can provide a free electron to trivalent iron atom, and form a pair of soliton electrons, the oxygen atoms in Fe_3_O_4_ form chemical bonds with hydrogen atoms in ammonia molecules, and the formation of hydrogen and oxygen bonds enhances the binding of ammonia molecules to Fe_3_O_4_ [34]. In addition to diffusion, CNTs can intermingle with Fe_3_O_4_ and enhance the adsorption of ammonia molecules by combining the advantages of both [17]. As a sensitive material, CNTs generally adsorb gas molecules and have no selectivity [35,36]. The addition of Fe_3_O_4_ can not only improve the adsorption of gas to the sensitive membrane because of the special bonding principle between ammonia and Fe_3_O_4_, but also produces a certain selectivity for ammonia gas. Figure 3 shows the principle of Fe_3_O_4_ binding to ammonia gas molecules.

In addition to the chemical bond, the magnetic catalytic effect of Fe_3_O_4_ can also promote the adsorption of gas molecules. The magnetic field produced by Fe_3_O_4_ as a magnetic material significantly affects the gas adsorption, which affects the gas adsorption rate of the chemical gas sensor. Ke and Shen studied this magnetic catalysis [37]. They found that the rate of the chemical reaction was affected by the external magnetic field; consequently, a theoretical model was established. The sensitivity was proportional to the exponential function of the square of the magnetic field, and was expressed in a simple equation (Equation (5)):

(5) $$S=M\times e^{\lambda \times B^{2}}$$

In the above equation, S is the sensitivity, B is the magnetic field intensity, and M and λ are the constants of the gas adsorption reaction. According to this equation, the greater the external field intensity, the faster the material reaction. Therefore, Fe_3_O_4_ can be used as a gas sensitive material to increase the reaction rate of gas adsorption, thereby improving the response speed of the sensor.

## 3. Experimental Section

### 3.1. Design and Manufacturing of the Sensor

**Figure 4:**
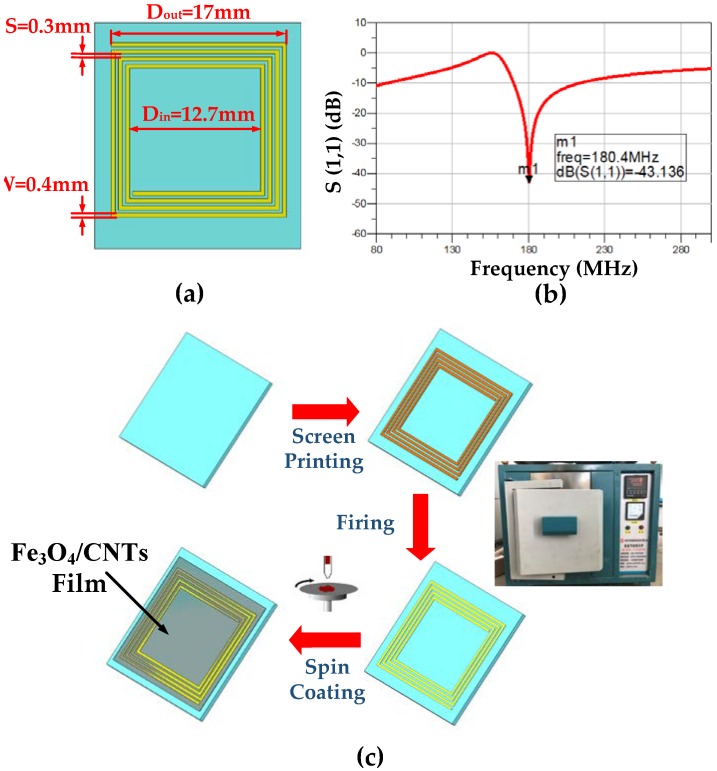
(**a**) Design of the LC (Inductance and capacitance resonant) wireless sensor. (**b**) Simulation results for the designed sensor. (**c**) Schematic of the fabrication procedure of the gas sensor.

The diagrammatic view of the LC wireless sensor’s structure is given in Figure 4a, which shows a square spiral inductor printed on a ceramic substrate. The parameters of the inductor were simulated using ADS (Advanced Design System 2009) for design optimization. The longest outer coil length L in the present work was 17 mm, the coil spacing S was 0.3 mm, and the coil width was 0.4 mm; the simulation results are shown in Figure 4b. A schematic of the fabrication procedure of the gas sensor is shown in Figure 4c. A screen printing plate was made first, and a silver paste was printed on the substrate in a screen-printing process. Then, the substrate was placed in a muffle furnace (BLMT-1800 °C, BLMT) to remove the impurities in the silver paste. The impurities in the silver paste were removed by burning in the muffle furnace after cooling, following which the configured Fe_3_O_4_/CNT suspension was coated on the inductor coil using spin coating. The setting speed of the spin coater (SPIN-51) was 500 revolutions/min, and the time was 25 s. After heating at 90 °C for 30 min, the thickness of the sensitive film was 130 nm.

### 3.2. Sensor Test

**Figure 5:**
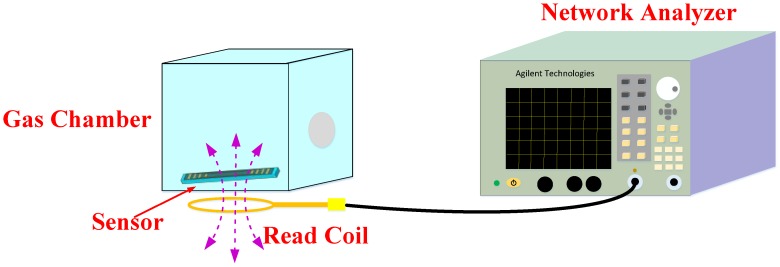
Schematic of the wireless gas sensing measurement setup.

Figure 5 schematically shows the wireless gas sensing measurement setup. First, the LC gas sensor was placed in a closed glass gas chamber. The coupling antenna was opposite the spiral inductor of the sensor and was connected to the network analyzer. The device was stationary for 200 s to stabilize the resonant frequency, and the ammonia gas was slowly delivered into the gas chamber (25 × 20 × 10 cm) using a syringe (1 mL); the measurement data were collected every 10 s. Experiments showed that after 300 s, the change in the resonant frequency of the sensor was no longer obvious and a fan was used to quickly discharge ammonia from the gas chamber; taking into account the recovery time of the sensor, the experimental time in the present work was 500 s. Immediately following the response time period, the gas chamber was opened and the ammonia gas in the gas chamber was allowed to completely escape. The time until the resonant frequency recovered to the initial state was recorded as the recovery time. The above process was repeated for different concentrations of ammonia, and each time the values of the resonant frequency and recovery time were registered.

## 4. Results and Discussion

**Figure 6:**
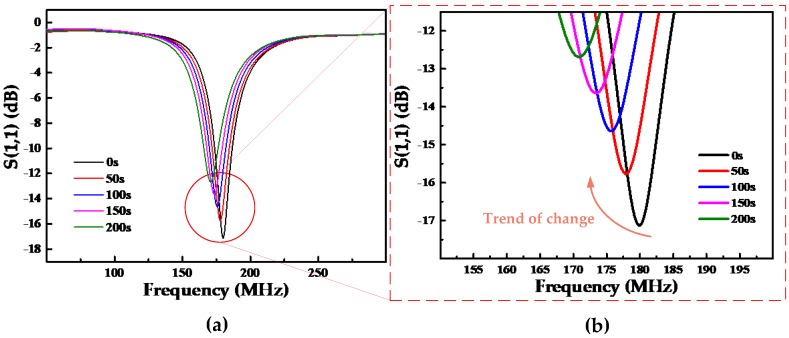
(**a**) Diagram of the change in the resonant frequency of the gas sensor at 40 ppm atmospheric ammonia at room temperature. (**b**) Amplified view into the trend of change plot in panel (**a**).

According to the previously proposed LC coupling model, the resonant frequency of the wireless passive gas sensor was measured using a coupling antenna and was displayed using the vector network analyzer in real time. Figure 6a shows the change in the resonant frequency of the gas sensor at 40 ppm atmospheric ammonia, at room temperature. Figure 6b is an amplified view of the encircled area in Figure 6a. The diagram suggests that the resonant frequency of the sensor gradually decreases with time, and the range of change is very large. 

The resonant frequency of the initial sensor was 180 MHz, which is lower than the 180.4 MHz frequency obtained in simulations, owing to the impedance discrepancy between the real sensor and the simulated one. At the same time, the results show that, in addition to the change in the resonant frequency caused by the change in capacitance, the S_11_ parameter also changes. This is owing to the electron migration that occurs when ammonia gas is adsorbed on the sensor. The transfer of some electrons from the sensitive film to the gas molecules increases the impedance of the sensor and decreases the S_11_ parameter. However, as mentioned before, S_11_ is affected by external influences. Therefore, the resonance frequency was chosen to indicate the concentration change.

**Figure 7:**
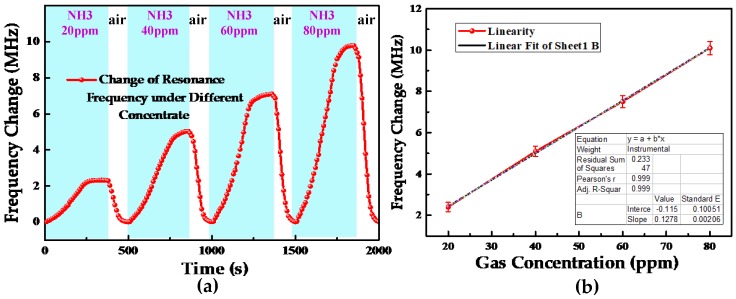
(**a**) Variation in the resonant frequency of the sensor for different concentrations of atmospheric ammonia. (**b**) Linearity curve of the sensor.

To study the relationship between the ammonia concentration and the resonant frequency of the sensor, for ammonia concentrations of 20, 40, 60, and 80 ppm, the change in the resonant frequency was tested for the same temperature and in the same environment. Figure 7a shows the variation in the resonant frequency of the sensor for different concentrations of atmospheric ammonia. The interval between every two data points in the curve is 10 s, and the curve shows that the LC gas sensor has good response and a short recovery time for different concentrations of ammonia, which proves that the designed sensor can be used for sensing ammonia concentration from 20 to 80 ppm. The recovery time is short, implying that the sensor can quickly recover to its original resonant frequency. As shown in Figure 7b, with the increase of ammonia concentration, the frequency change of the sensor increases linearly. This can be explained as follows: As the concentration of ammonia increases, more ammonia molecules are adsorbed onto the surface of the sensor gas-sensitive film; consequently, more electrons migrate, the capacitance of the sensor increases, and the variability of the sensor’s resonant frequency also increases. The frequency change of the sensor is greater than 2 MHz at 20 ppm ammonia concentration. 

**Figure 8:**
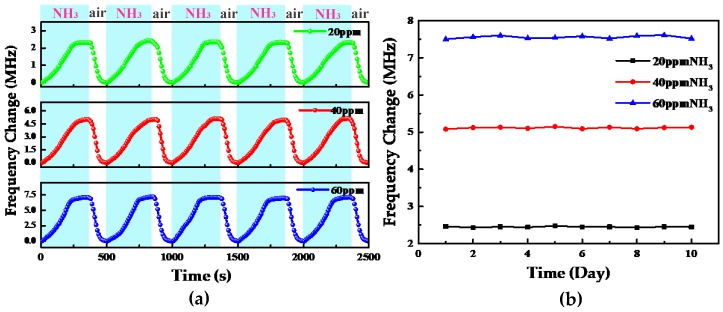
(**a**) Repeatability of the gas sensor exposed to 20, 40, and 60 ppm of atmospheric ammonia, at room temperature. (**b**) Long-term stability of the gas sensor exposed to 20, 40, and 60 ppm of acetone gas.

Figure 8a shows the repeatability of the same gas sensor exposed to 20, 40, and 60 ppm of atmospheric ammonia at room temperature. For ammonia concentrations of 20, 40, and 60 ppm, the response and recovery performance of the sensor are very good. The repeatability test of the sensor was done for five response recovery cycles. The results show that the repeatability of the sensor is good, but the response and recovery are still slightly different across the different cycles. This may be because as a chemical gas sensor, the gas sensitive material will deteriorate gradually, and during operation at high frequencies, the charge distribution of the coupled antenna and sensor coil is close to the surface of the conductor owing to the skin effect [38]. When the frequency changes, the distribution of the charge changes as well. In reality, it is not possible to recover exactly the same experimental conditions; thus, charge transfer differs slightly across experiments, resulting in slight differences between the results of different test cycles. In addition, Fe_3_O_4_/CNTs film can be considered as a lossy sensitive film; a possible reason for the difference of the resonant frequency in each test cycle is the loss of the film. To determine whether this loss has a significant impact, we performed a 10-day stability test of the sensor’s gas-sensing performance. The results of this test are shown in Figure 8b. The entire test lasted 10 days and was performed for ammonia concentrations of 20, 40, and 60 ppm. The test results showed that the readings of the gas sensor did not change significantly during the testing process, indicating that the repeatability of the gas sensor was good, and the stability of the gas sensing material was good.

**Figure 9:**
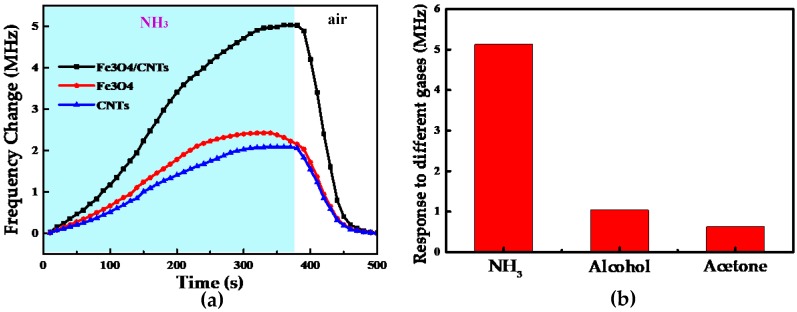
(**a**) Responses of different gas sensitive films to ammonia. (**b**) Response of the proposed sensor to different gases.

As mentioned before, Fe_3_O_4_/CNTs can combine the advantages of the two respective pure materials to improve the response speed of the sensor and to increase the response to ammonia compared with other gases. To confirm this conjecture, the same sensor was made in the same process and coated with CNTs, Fe_3_O_4_, and CNTs/Fe_3_O_4_ sensitive membranes, respectively, and the test was conducted for the same concentration of ammonia. As shown in Figure 9a, for the 40 ppm concentration of atmospheric ammonia, the response speed of the sensor with Fe_3_O_4_/CNTs as a sensitive film was significantly higher compared with the other two, which confirms that the previous conjecture is correct. In this work, we also considered the same concentrations of alcohol (g), acetone (g), and ammonia (g), and tested the developed sensor on these. The results of this experiment, shown in Figure 9b, suggest that the response of the sensor to the 40 ppm concentration of atmospheric ammonia is much stronger than those to the other gases. Therefore, the sensitive membrane used in the LC gas sensor designed in the present work has a higher response speed and selectivity to ammonia than individual CNTs and Fe_3_O_4_. 

Table 1 summarizes some of the existing research results on ammonia sensing. It provides a comparison between the sensing materials, response recovery time and the minimum ammonia concentration of the sensors. It can be seen that the sensors in this paper have a low detection limit.

## 5. Conclusions

In this paper, a wireless passive gas sensor was designed and manufactured to detect ammonia concentration. The gas sensing material used by the sensor was Fe_3_O_4_/CNTs. The coupling principle of the LC gas sensor was analyzed, and the sensitive mechanism of the sensitive material to ammonia gas was expounded. The tests of the sensor on different concentrations of ammonia gas showed that the sensor has a better response speed to the ammonia gas, and the repeatability and stability of the sensor were tested as well. These results suggest that the sensor is stable. Compared with individual CNTs- and Fe_3_O_4_-based sensors, the test results show that the gas sensing performance of the proposed sensor is better than that of the sensors that use CNTs and Fe_3_O_4_ as sensitive films, and the reaction to ammonia is more intense compared with those using other gases. Therefore, the sensor is characterized by a good performance and has good application prospects.

## Figures and Tables

**Table 1:** Comparison of response of the developed sensor in this work with those of other materials-based gas sensors to ammonia at room temperature.

Materials	NH_3_ Concentration	Response Time	Recovery Time	Reference
Fe_3_O_4_/CNTs	20 ppm	290 s	100 s	This work
PANI/HCSA	100 ppm	20 s	80 s	[39]
RGO-A (aniline reducing)	20 ppm	1200 s	300 s	[40]
single ZnO-T−CNT	100 ppm	20 s	420 s	[41]
MWCNT/PEDOT:PSS	30 ppm	1200 s	300 s	[42]
Natural Carbonized Sugar	100 ppm	50 s	42 s	[43]
